# Home Enteral Nutrition in Singapore’s Long-Term Care Homes—Incidence, Prevalence, Cost, and Staffing

**DOI:** 10.3390/nu11102492

**Published:** 2019-10-17

**Authors:** Alvin Wong, P Marcin Sowa, Merrilyn D. Banks, Judith D. Bauer

**Affiliations:** 1 School of Human Movement and Nutrition Sciences, The University of Queensland, St Lucia, QLD 4072, Australia; merrilyn_banks@health.qld.gov.au (M.D.B.); j.bauer1@uq.edu.au (J.D.B.); 2 Department of Dietetic & Food Services, Changi General Hospital, Singapore 529889, Singapore; 3 Centre for the Business and Economics of Health, University of Queensland, Woolloongabba, QLD 4102, Australia; m.sowa@uq.edu.au; 4 Royal Brisbane and Women’s Hospital, Herston, QLD 4029, Australia

**Keywords:** home enteral nutrition, tube feeding, dysphagia, long-term care, cost

## Abstract

*Introduction*: Data on home enteral nutrition (HEN) in long-term care facilities (LTCF) in Singapore is scarce. This study aims to determine the prevalence and incidence of chewing/swallowing impairment and HEN, and the manpower and costs related. *Methods:* A validated cross-sectional survey was sent to all 69 LTCFs in Singapore in May 2019. Local costs (S$) for manpower and feeds were used to tabulate the cost of HEN. *Results:* Nine LTCFs (13.0%) responded, with a combined 1879 beds and 240 residents on HEN. An incidence rate (IR) of 15.7 per 1000 people-years (PY) and a point prevalence (PP) of 136.6 per 1000 residents were determined for HEN, and an IR of 433.0 per 1000 PY, with PP of 385.6 per 1000 residents for chewing/swallowing impairment. Only 2.5% of residents had a percutaneous endoscopic gastrostomy (PEG). The mean length of residence in LTCF was 45.9 ± 12.3 months. More than half of the residents received nasogastric tube feeding (NGT) for ≥36 months. Median monthly HEN cost per resident was S$799.47 (interquartile range (IQR): 692.11, 940.30). Nursing costs for feeding contributed to 63% of total HEN costs. *Conclusions:* The high usage and length of time on NGT feeding warrants exploration and education of PEG usage. A national HEN database may improve the care of LTCF residents.

## 1. Introduction

Enteral nutrition is a life-saving and life-sustaining therapy for patients who are unable to obtain adequate nutrition via the oral route. One of the main indications for enteral nutrition is dysphagia, which is the impairment of swallowing. The prevalence of dysphagia ranges from 7% to 40% in long-term care facilities (LTCF) [1] and the majority of patients with dysphagia will likely require home enteral nutrition (HEN) at some point in their life. The prevalence of dysphagia has also been shown to be higher in LTCF as compared to community settings [2]. Other indications for HEN include the need to improve the nutritional status of patients who have suboptimal oral intake, and gastrointestinal problems such as obstruction and malabsorption [3]. 

The yearly prevalence of HEN was estimated at 463 per million population in the United States with an incidence of 163 to 360 per million population in the United States and Europe in the 1990s [4,5]. An eight-fold increase was reported recently, with an estimated prevalence of 1385 per million population [6]. In the United Kingdom, the latest British Artificial Nutrition Survey (BANS) reported a 5% increment in HEN between 2009 and 2010. Between 2% and 34% of residents in nursing homes in the United States are on HEN [7]. Except for patients with dementia [8,9] and people on end-of-life care [10], the use of HEN is both clinically effective and cost-effective in populations such as patients with pressure ulcers [11], and those that switched from blenderized diet to commercial feeds [9].

There are currently no statistics or data on patients with dysphagia or on HEN in Singapore. Data are also limited in the Asia Pacific region, as shown by a recent survey on HEN reimbursement and practices [12]. A Japanese study reported a prevalence of 7.4% to 11.6% of LTCF residents on HEN [13]. Amongst the types of HEN delivery methods, the majority of patients were on nasogastric tube feeding (NGT) in the Asia Pacific [12], although actual figures were not available as most countries do not have a national census. Prevalence and incidence data on the use of percutaneous endoscopic gastrostomy (PEG) in LTCFs in the Asia Pacific is limited, with a 2.8% prevalence rate in a Taiwanese study [14], and another Japanese study estimating a prevalence of 89,096 PEG patients in 2011 [15].

The majority of patients on HEN reside in LTCFs, such as nursing homes in Singapore. There are currently 69 nursing homes (74 in total excluding new homes that are not fully operational at the time of manuscript preparation and dementia-care homes) in Singapore with a total of 15,205 beds, and the cost of stay ranges from S$1200 to S$3500 per month (before subsidy) [16]. The structure of LTCFs in Singapore is as described in Appendix A. Under the Ministry of Health’s Enhanced Nursing Home Standards (ENHS) in 2014, all nursing homes are required to a) perform nutritional screening for all residents once every six months, b) refer the resident to a qualified dietitian for an individual assessment if there is weight loss/poor oral intake/any conditions identified which require nutritional intervention (HEN inclusive), and c) hire a qualified dietitian to monitor the resident’s nutritional status and dietary intake [17]. There are no reports or findings if the new standards are fully implemented and the efficacy of the interventions. 

The cost for HEN in LTCFs has been reported to be higher than for HEN patients residing in their own homes [9,18], but there is currently no cost data available for the Singapore population. Recently, subsidies were implemented for parenteral nutrition (acute care and home), but there has not been any plan for HEN subsidy, although nursing homes can purchase a limited variety of enteral feeds through a national Group Purchase Order (GPO) at discounted prices. With an ageing population in Singapore, where the proportion of residents aged 65 years and over has increased from 8.5% to 13.0% between 2007 and 2017 [19], it is likely that the number of patients/residents on HEN will increase in the near future, as shown by data from Europe/the US [6], and manpower needs and costs are likely to substantially increase as well.

In order to determine the potential costs and cost-effectiveness of HEN for the local LTCFs (specifically nursing homes), the availability of local data is essential. As such, the aims of this study were to determine the

(1) Prevalence and incidence of swallowing impairment and HEN,
(2) Types of enteral feeding methods/nutritional feeds used, and the local cost data of administering HEN,
(3) Descriptive profiles of LTCFs and the nursing allocation for managing patients on HEN, and
(4) Demographics of patients/residents residing in LTCFs.

## 2. Methods

A cross-sectional survey was sent out to 69 LTCFs in Singapore to determine the prevalence and incidence of HEN and swallowing impairment, as well as the demographic details of local LTCFs and residents, the type of HEN delivery and feeds used, and information on nursing manpower and HEN administration. We excluded dementia-care homes and new LTCFs that were not fully operational at the time of survey.

An original, purpose-built survey was developed by the investigators with the aim of increasing participation rates. A validation panel consisting of five experts in the field was invited to determine the face and content validity of the questionnaire based on the set of evaluation criteria, as described by Neuman [20], Berdie et al. [21], and Dillman [22]. The panellists reviewed the questionnaire for face and content validity via an online structured reporting form (Google Form, Appendix B) developed by the researchers. Validation comprised of the following aspects: A) clarity, comprehensiveness, and appropriateness of the questions/responses; B) usefulness of the survey; C) layout of the form and sequence of the questions; D) face and content validity; and E) time needed to complete the survey. 

The LTCFs included private as well as those by voluntary welfare organizations or governmental agencies, to provide a mix of resident populations. As residents in Singapore are assigned randomly to nursing homes based on the availability of beds and family’s/individual’s preferred location at the time of admission, sample populations were assumed to be broadly representative of the population of interest. Data were collected in May 2019. Dietitians at the LTCFs were asked to complete the questionnaire. If an in-house dietitian was not available, a registered nurse or medical staff were requested to complete the questionnaires. Exclusion criteria included LTCFs without patients on HEN, specific dementia-care homes, and new LTCFs that were not in full operation at the time of the survey. 

For the purpose of simplifying the survey for the respondents, we considered residents who required a modified texture diet or a modified fluid viscosity based on the International Dysphagia Diet Standardisation Initiative (IDDSI) as proxies for swallowing/chewing impairment [23]. We determined the incidence rates per 1000 person-years (PY) for new feeding tube insertions and swallowing/chewing impairment by the following formula: Number of Incident Cases Accumulated Time Under Risk.

Point prevalence was calculated as the number of residents with swallowing impairment or on tube feeding per 1000 residents. The reference population was based on the total population of the participating LTCFs.

The cost of nutritional feeds was calculated using the recommended retail price (July 2019) and also based on the available discounted GPO prices, with a 30% and 45% discount (personal communication) performed on additional feeds where GPO prices were not accessible. GPO prices are renegotiated once every five years and hence not susceptible to short-term fluctuations. Nursing costs were determined using the median monthly pay of S$1800 (tax-adjusted, the year 2018) [24] of enrolled nurses, extrapolated to the total time spent performing enteral feeding over the entire day (based on the survey’s median time reported). Enrolled nurses’ pay was chosen as most enteral feeding procedures in LTCFs and hospitals are performed by them. All costs were reported in Singapore Dollars.

## 3. Statistical Analysis

Statistical analysis was performed using SPSS version 25 (IBM Corp., Armonk, NY, USA) software package. Values were reported as mean (±S.D.) or median with interquartile range (IQR). Independent-samples *t*-test or nonparametric tests (Mann–Whitney U and chi-square tests) were used to determine differences between groups. A *p*-value of <0.05 was considered statistically significant.

## 4. Ethics Approval

Ethics approval was obtained from The University of Queensland (Approval number 2018001212). Ethics approval was not required from Sing Health Centralised Institutional Review Board (Singapore) as studies that do not have personal data recorded or intervention are considered as surveillance programs and hence not under purview.

Informed consent to participate in the study was taken by panellists consenting and completing the online structured reporting form and submitting it to the researcher for the survey validation. Implied consent was taken as participants who agreed to participate will return the completed survey forms via email.

## 5. Results

Profiles of the LTCFs are described in Table 1. Nine LTCFs responded to the survey (participation rate 13.0%) with a combined 1879 beds, equivalent to 12.4% of total nursing home beds in Singapore. A 93.5% bed occupancy was reported during the month of the survey. Fourteen LTCFs declined to participate, forty-three LTCFs did not respond to the request, one LTCF did not return the survey questionnaire after the closing date of survey, one LTCF was not fully operational, and one LTCF was a specific dementia-care home. Forty-four percent (44%) of the respondents for the survey were dietitians, and the remainder were nursing managers or senior registered nurses. 

The majority of LTCFs have outsourced dietetic and speech/swallowing therapy services, with a median dietetic review frequency of 0.5 times per month (IQR: 0.3, 1.5) and median speech/swallowing review frequency of 1.0 time per month (IQR: 0.0, 6.0), in LTCFs that do not have a full-time dietitian or speech therapist. Nutritional screening is performed for all patients on admission with 77.8% of LTCFs performing repeated nutritional screening at three- or six-monthly frequencies. Speech/swallowing therapy was not available in 44.4% of the LTCFs surveyed. 

A total of 585 residents, with 45 new cases identified, required a modified texture diet or modified consistency fluid. The overall incidence rate of residents with swallowing or chewing impairments (excluding residents on HEN) was 433.0 per 1000 PY, with a point prevalence of 385.6 per 1000 residents. This increased to an incidence rate of 447.3 per 1000 PY and point prevalence of 469.6 per 1000 residents when residents on HEN were included.

Only two new feeding tube insertions were reported for all the LTCFs surveyed, with a total of 240 residents on HEN. The overall incidence rate of new feeding tube insertions was 15.7 per 1000 PY. The point prevalence of HEN was 136.6 per 1000 residents. The majority of the residents on HEN received feedings via a nasogastric tube (NGT) (97.5%), with the rest on percutaneous endoscopic gastrostomy (PEG) (2.5%). No residents were identified with other types of feeding tubes. Bolus feeding was the only method of administration in all nursing homes. 

The nursing practices and workforce is presented in Table 2. Nursing aides make up the majority of nursing care professionals in this setting. Enrolled nurses were allowed to perform tube feeding for patients in all nursing homes. Half (55.6%) of the nursing staff received less than one hour of nutritional training per annum, but 66.7% felt that the training was sufficient. Two-thirds of the respondents were uncertain if feeding pumps would be beneficial or were certain that feeding pumps were not suitable for use in nursing homes.

The nutritional profile of the residents is presented in Table 3. The mean age of a nursing home resident was 74.7 years. Female residents were generally older and of lower body weight when compared to male residents in nursing homes. The majority of the residents received standard polymeric feeds, and a quarter of residents (24.4%) were receiving diabetes-specific enteral nutritional feeds. Overall, including the fibre-containing diabetes-specific feeds, 28.8% of residents were receiving fibre in the HEN regimen. A small proportion (4.6%) of residents required specialized feeds such as renal and peptide-based feeds. Residents received a mean energy intake of 29.1 kcal/kg and a protein intake of 1.2 g/kg/day via HEN. 

No differences were found between genders in terms of weight-based energy and protein intake, length of time on HEN, and residency time in nursing homes. 

There were also no differences across all nutritional intake variables when compared between outsourced versus in-house dietitians, frequencies of dietitian visits, and types of LTCFs (private versus public/not-for-profit). 

The mean length of time a resident spent residing in the nursing home was approximately 46 months, with a maximum of 228 months. More than half of the residents on HEN received nasogastric tube feeding for more than 36 months. Only 3.3% (*n* = 8) of the residents were on supplementary tube feeding. 

The median cost of HEN per resident per month is depicted in Table 4. The cost of HEN per resident per month ranges from S$715.85 to S$856.19. When feeds were costed at recommended retail prices, there were no differences between the monthly cost of HEN for the different feed types. At 30% and 45% discounted Group Purchase Order prices (applicable only to a selected few standard feed types), ready-to-use feeds were significantly more expensive than powdered feeds (*p* < 0.001), and specialized feeds more expensive than standard feeds (*p* < 0.001). Nursing costs for feeding contributed to 58% of total HEN costs. 

## 6. Discussion 

This is the first HEN survey conducted in Singapore to determine the incidence and prevalence of tube feeding and swallowing/chewing impairment in LTCFs, as well as the various factors contributing to HEN. We identified an incidence rate of 433.0 per 1000 PY, with a point prevalence of 385.6 per 1000 residents (38.6%) for swallowing/chewing impairment, equivalent to 5482 LTCF residents in the country. This is comparable with the recent Nutrition Day data (2007–2014) where 24.8% of LTCF residents were diagnosed with dysphagia, and 35.1% reported chewing difficulties [25].

The HEN incidence was identified to be 15.7 per 1000 PY with a point prevalence of 136.6 per 1000 residents (13.7%), comparable to international figures [8,9]. We estimate that there are approximately 1942 LTCF residents on HEN in Singapore (based on estimated bed occupancy rate of 93.5%). In our survey, only 2.5% of residents on HEN received PEG feeding, and the majority of the residents have been receiving HEN via NGT for more than 36 months. Current ESPEN guidelines recommend that “Older patients expected to require enteral nutrition for more than four weeks or who do not want or tolerate a nasogastric tube should receive a percutaneous gastrostomy/PEG” [26]. Patient and medical staff perspectives play a role in determining if a PEG is inserted. Lin et al. [27] conducted a face-to-face interview with 607 patients, and some of the common themes found for refusing a PEG were that they were “too old to suffer from an operation”, worried about infection/leakage after the procedure, and that they wished to keep their body’s integrity. Compounded with the traditional Asian belief that a dying individual should not be deprived or starved of food in their last living days, a nasogastric tube is generally the preferred choice of tube feeding. [28] 

The relative ease of inserting NGT may account for its preferred use as well, as shown in this survey, where the majority of nursing staff take less than 15 min for a change of tube. This is in comparison to a PEG insertion which requires residents to be admitted to a hospital facility for endoscopic procedures. The lack of choice because of insufficient provision of information, lack of national guidelines, constraints of resources, and influences from family and healthcare professionals have also been reported as barriers to PEG [29]. Another possible reason could be the fear of complications. Compared to NGT feeding, PEG feeding is effective and safe, with clear benefits in patients with stroke through the reduction of treatment failures and risks of gastrointestinal bleeding [30]. PEG also provided higher amounts of feeding delivery and increased albumin concentration [31]. However, recent studies have shown that mortality risk increases with PEG feeding in the elderly and those with dementia [32,33,34,35].

The surveyed residents’ length of time on HEN in this survey population is higher than internationally reported results. More than 50% of residents in our survey population have been fed via a feeding tube for more than 36 months, similar to another Asian study [14], but a high figure in comparison to a German study where a median length of time on HEN was reported to be 17.9 months (range of 5.6–26.5 months) [36]. Another Italian study reported the median length of time on HEN as 6.5 months (196 days, range 82.5–788 days) [37]. The differences in length of time could be due to the different LTCF populations between the different countries, or it could be due to local practices where tubes are readily inserted in LTCF residents and the lack of follow-up to wean off tube feeding.

It takes approximately 38 days after a stroke before a patient can resume some form of oral diet [38]. Given the priority that hospital systems place on reducing the length of hospital stay, a majority of patients placed on NGT feeding may be discharged back to LTCFs or homes with the tube in situ, while waiting for an outpatient speech therapist review that may only be scheduled months after discharge. This coupled with the lack of speech/swallowing therapy services in LTCFs (median visit frequency of once per month in our survey), residents may be on prolonged HEN even if they have the potential to resume an oral diet. Not surprisingly, the majority of feeding tubes are inserted in the acute care settings [39,40,41]. This may explain our survey’s low incidence but high prevalence of tube feedings, indicating that feeding tubes are likely inserted in hospitals before transfer to LTCFs. 

Other potential problems that residents on HEN faced include malnutrition. Risk factors for malnutrition in LTCFs, as demonstrated by the NutritionDay survey, include elderly age >90 years, immobility, dementia, and dysphagia. Volkert et al [36] reported 57.7% of nursing home tube-fed residents were malnourished. We were unable to determine the malnutrition rates in this survey as the main aim was to establish the incidence and prevalence of tube feeding and swallowing/chewing impairment in Singapore. The mean caloric intake (29.1 kcal/kg) of residents in our survey were slightly lower than the recommended 30kcal/kg by the ESPEN guidelines on clinical nutrition and hydration in geriatrics [26]. This may be a potential risk for malnutrition as some of the HEN population in LTCFs may not be receiving adequate intake or dietetic review. Although the mean protein intake met the recommended intake of 1.2g/kg for the elderly, it may not be sufficient for those who have chronic diseases, pressure injuries, or are malnourished, which represents a significant proportion of LTCF residents.

Residents on HEN also require greater nursing and allied health professional care. The ENHS or Private Hospitals and Medical Clinics (PHMC) Act [42] currently does not stipulate the number of hours that a dietitian or a speech/swallow therapist is required to spend with each resident/patient in the nursing home. We were unable to conclude if an LTCF with more dietetic consultations has a higher proportion of residents meeting nutritional targets. The likely reason is that dietetic review hours are insufficient to begin with, even in LTCFs with a full-time dietitian. Based on the local practices for outsourced dietetic services, most LTCFs only have one dietetic visit per month (4 to 6 h per session) for a 100 to 200 bed LTCF. 

A recent American study reported a mean 0.53 Full-Time Equivalent (FTE) dietitian per 100 residents in LTCF [43]. The Canadian long-term care services have a stipulated dietetic review time of 30 min per resident per month, equivalent to 12 h per week in a 100-bed LTCF [44]. This is equivalent to a minimum of 0.5 FTE dietitians in a typical 200-bed local LTCF, which is unattainable at current ENHS recommendations of one dietitian visit (no minimum hours requirement) every six months. We were unable to identify any guidelines regarding recommended speech/swallowing therapy review hours for LTCF, but it is likely to be higher than dietetics, given the additional time required for swallowing assessment. Although no research has explored the impact of speech/swallowing therapy or dietetic staffing on long-term outcomes of nursing home residents, research on direct-care staffing has suggested a positive relationship between staffing levels with quality [45].

The PHMC Act [42] also requires at least one registered nurse contactable at all times. We were unable to determine if staffing is sufficient for residents on HEN due to the complexity of nursing to patient ratio requirements in Singapore. In general, the nursing to patient ratio is dependent on which the category of care the resident requires (in ascending order): Category 1, 1:30; Category 2, 1:8; Category 3, 1:4; and Category 4, 1:2. Residents with high care needs, such as the bedbound HEN residents, are usually in Category 4. However, these ratios are overall norms and not per shift norms, that is, if a nursing home has 40 Category 4 residents, the LTCF shall have a total number of 20 care staff. The LTCF is given the flexibility of rostering these care staff to provide care for the residents over 24 h.

We also identified an initial nutrition screening rate of 100% on admission to a nursing home, which is not unexpected as the ENHS stipulates it. However, it appears that repeated screenings were not carried out consistently across all the nursing homes (22%). This is comparable to international data, where 24% of nursing homes do not perform routine weighing/screening [46]. Given the upper limit of length of residence in nursing homes is 228 months (mean of 45 months), it is imperative that regular nutritional screening for this group of individuals is performed. With the implementation of electronic medical documentation in LTCFs in Singapore, this issue will most likely be resolved as screening schedules can be set electronically.

Data from our survey indicated that it costs S$871.43 to feed a resident on a full enteral nutrition regimen per month, inclusive of nursing costs to administer the feeds. There is no official data on food or HEN costs in nursing homes in Singapore. Food budgets in overseas (USA, UK, Canada, and Australia) aged care facilities range from S$8 to S$22 per day [47]. In a recent nongovernmental organization’s report on the economics of Singapore nursing home care, the estimated cost for nursing home care in nonprivate homes was S$106.10 per day, and approximately $15 was for food [48]. The daily cost of nutritional feeds in our study was S$6.63 to S$11.64, which is lower than the budgeted food cost. However, some residents or their family members may be required to pay additional costs (through the LTCF’s supply chain or external retailers) for the use of higher-priced feeds (e.g., high calorie and protein, renal, diabetes-specific, and peptide-based feeds) where residents are penalized for their underlying comorbidities. This is a substantial cost that the government should consider when providing subsidies for LTCFs as more than 50% of residents in our survey remained on HEN for at least three years. 

Although GPO prices available to LTCF are generally lower than recommended retail prices, the disparity between specialized/energy-dense feeds, and powdered/standard feeds is still apparent. This may be due to the push by pharmaceuticals to encourage the use of standard/powdered feeds, which is supported by our data of higher use of standard/powdered feeds. One of the reasons we postulate is that powdered feeds are generally cheaper to manufacture due to less packaging involved and longer storage life. The other possible reason is that other facilities such as acute-care (acute and community hospitals) tend to use more ready-to-use feeds due to convenience, inadequate manpower, and reduced contamination risks from feed preparations, therefore there is a need to push for higher powdered feeds’ usage/sales in LTCFs. The proportion of patients who require specialized and energy-dense feeds may also be lower due to the limited information available for recommending the use of such feeds in HEN populations [26]. However, the low use of fibre-containing feeds is an issue of note, as fibre intake may be beneficial for both diarrhoea and constipation in HEN populations [49].

## 7. Limitations

One of the limitations of the study is the proportion of nursing homes that responded to the survey rate. The correspondence rate of 13.0% in our survey is similar to another study in Germany (11.5%) [41]. One of the reasons for the relatively low uptake of the survey is that some homes were unwilling to participate out of fear of less than optimal results when compared to other nursing homes [46]. We sought to alleviate the concerns by indicating on the survey forms and in the invitation emails that names of participating nursing homes would not be revealed. 

Another possible limitation is the selection bias of nursing homes as larger nursing homes that are better funded and organized may be more willing to participate in the survey. This may be less likely an issue, as participating nursing homes were well distributed in terms of type (private and public) and size (bed numbers ranged from 89 to 386), and more likely had better nursing, HEN data, and weight records. Other limitations that relate to data collection include decreased reliability as data were collected by nursing home staff and not trained research coordinators or investigators. 

However, the invited validation panel comprised of experienced nursing home dietitians who reviewed the survey for clarity and face and content validity before distributing the surveys. The 56% public/VWO/not-for-profit LTCF to 44% private LTCF ratio for our survey is likely to be representative of the population as it is comparable to the public/VWO/not-for-profit to private LTCF ratio in the country (Refer to Table A1 in Appendix A). The data obtained is also likely generalizable to the country’s LTCF population as all LTCFs’ clinical and manpower allocation practices have to comply with the Ministry of Health’s Enhanced Nursing Home Standards [17] and the Private Hospitals and Medical Clinics Act [42].

There was also difficulty in obtaining the actual length of time on HEN data, as nursing homes generally do not record the first date that a resident receives an enteral feeding tube insertion. Additionally, some residents may have moved between nursing homes, which is common as residents may not be allocated their first choice on initial placement (e.g., somewhere nearer to their family home) and admitted to interim nursing homes. We were also unable to obtain weight histories, as the feedback from the validation team was that excessive data collection might result in a lower participation rate. The main reasons that nursing homes gave for declining participation in the survey were also related to a lack of manpower or that it was too much work for the staff. 

For the same reasons as above, we had to use the prescription of modified texture diet and fluid viscosities as proxies for dysphagia diagnosis. It was also difficult to differentiate between dysphagia and chewing difficulties as dysphagia is not always recorded as a diagnosis from a speech/swallow therapist or medical history in LTCF residents in Singapore. Residents in LTCFs may be on a modified diet because of poor dentition instead of dysphagia, as edentulousness is prevalent in approximately half of these residents [50]. Hence, actual incidence and prevalence figures may be lower than the levels reported in our survey.

The costing methodology may also be too simplistic, as the Ministry of Health in Singapore does not release the GPO price of nutritional feeds available, and pharmaceutical companies do not disclose contract prices due to contracture agreements and conflict of interests. As such, we used the recommended retail price and applied discounted prices of 30% to 45% (personal communication) to account for the range of costs involved in HEN. As bolus feeding is the primary method used in LTCFs, the cost components only involve manpower and perishable (feeds) and disposable (feeding tubes and syringes) items, which reduces the inaccuracies involved in complex feeding methods such as continuous pump feeding that involves additional costs in equipment, maintenance, training, and feeding peripherals.

## 8. Conclusions

The incidence and prevalence of HEN in nursing homes are similar to other developed countries. There is an exceptionally high usage of NGT in the homes, and majority of the residents are on NGT for more than three years, indicating the need for educating family members and LTCF staff to explore the use of PEG instead to improve quality of life outcomes in the nondementia group of residents. The lack of dietetic and speech/swallowing therapy support will also need to be addressed, as it is likely that residents who require HEN and residents who may have dysphagia will increase as the ageing population increases. Future governmental policies and enhancement to guidelines will also be required to optimise the level of staffing for dietitians and speech/swallow therapists in LTCFs.

There is a need for a national registry of patients and residents on HEN to allow healthcare systems to track the progress of HEN patients and improve communication of the care of residents between hospital and LTCFs/caregivers, to reduce the use of unnecessary NGT feedings in residents. Further research will need to identify the quality of life of HEN patients and long-term outcomes for those on long-term NGT.

## Figures and Tables

**Table 1:** Long-Term Care Facilities’ Profile.

Nursing Home Descriptors	Frequency	% or IQR
Number of Nursing Homes	9	13.0
Number of Beds		
Total	1879	12.4
Minimum	89	−
Maximum	386	−
Total Number of Residents at Survey Period		
Total	1757	−
Minimum	89	−
Maximum	340	−
Types of Nursing Home		
Private	4	44.4
Public/VWO/Not-For-Profit	5	55.6
Dietetic Service		
In-House Dietitian	2	22.2
Outsourced Dietetic Services	7	77.8
Frequency of Dietetic Services Per Month for Homes without an In-House Full Time Dietitian	0.5	IQR: 0.3,1.5
Dietetic Referral Methods		
Automatic Referral with Open Date Reviews	6	66.7
Automatic Referral with Fixed Reviews	3	33.3
If Hospital Memo or Transfer Letter Requested	5	55.6
Nursing Home Medical Staff Requested	6	66.7
Family Requested	4	44.4
Nursing Staff Requested	6	66.7
Nutritional Screening Performed for All Patients	9	100
Nutritional Screening Repeated		
Yes	7	77.8
No	2	22.2
How Often is Nutritional Screening Repeated		
3-Monthly	3	33.3
6-Monthly	4	44.4
Not Repeated	2	22.2
Speech/Swallowing Therapy Service		
In-House Speech Therapist	0	0
Outsourced Speech Therapy Services	5	55.6
No Speech Therapist Services	4	44.4
Frequency of Speech/Swallowing Therapy Services per month	1.0	IQR: 0.0, 6.0
Required Modified Texture Diet or Modified Fluid Viscosities		
New Cases Per Month	45	−
Total	585	−
Required Tube Feedings (HEN)		
New Cases Per Month	2	−
Total	240	−
Types of Tube Feedings		
Nasogastric	234	97.5
PEG	6	2.5
Method of Feeding		
Bolus	240	100

Total number of nursing homes at time of survey = 69. Total number of nursing home beds in Singapore in 2018 census = 15205. HEN: Home Enteral Nutrition; IQR: Interquartile range; VWO: Voluntary Welfare Organization.

**Table 2:** Nursing Practices and Manpower in Nursing Homes.

Nursing Practices	Frequency	% or Range
Feeding Methods Available in Nursing Homes		
Bolus	9	100
Continuous via Pump	0	0
Continuous via Gravity Drip	0	0
Frequency of Nasogastric Feeding Tube Changes		
Fortnightly	1	11.1
Monthly	7	77.8
Quarterly	1	11.1
Time Taken to Change a Nasogastric Tube		
<10 min	1	11.1
10 to <15 min	6	66.7
>15 min	2	22.2
Can Special Feeds be Ordered for Patients		
Yes	8	88.9
No	0	0
Unsure	1	11.1
Who Performs Enteral Feeding		
Registered/Staff Nurse	8	88.9
Enrolled Nurse	9	100
Nursing Aide	8	88.9
Time Taken to Perform One Enteral Feeding		
10 min	2	22.2
15 min	6	66.7
20 min	1	11.1
Should Pumps be Made Available to Nursing Homes		
Yes	3	33.3
No	3	33.3
Unsure	3	33.3
Registered/Staff Nurses		
Number in Nursing Home	10.0	IQR: 6.5, 13.0
Number per Shift	3.0	IQR: 2.0, 5.0
Nursing to Patient Ratio per Shift	2.2:100	Minimum: 1.2:100
		Maximum: 4.1:100
Enrolled Nurses		
Number in Nursing Home	8.0	IQR: 7.5, 12.0
Number per Shift	3.0	IQR 2.5, 4.0
Nursing to Patient Ratio per Shift	1.9:100	Minimum: 0.2:100
		Maximum: 3.3:100
Nursing Aides		
Number in Nursing Home	45	IQR: 14, 54.5
Number per Shift	8.0	IQR: 3.5,15.0
Nursing to Patient Ratio per Shift	5.3:100	Minimum: 0.8:100
		Maximum: 10.3:100
Number of Hours of Nutritional Support Training Nursing Staff Receives Annually		
<1 h	5	55.6
1 to <3 h	3	33.3
3 to <5 h	1	11.1
Are Nursing Staff Provided with Adequate Training in Nutritional Support		
Yes	6	66.7
No	3	33.3

IQR: Interquartile range.

**Table 3:** Nutritional Profile of Residents in Nursing Homes on Tube Feeding.

Profile of Residents on Tube Feeding	All Residents	Female	Male	*p*-Value	95% CI of Difference
Gender	240	113	127		
N (%)	100	47.1	52.9	0.401	−
Age (years ± S.D.)	74.66 ± 13.2	78.9 ± 12.6	70.9 ± 12.6	<0.001	4.77, 11.36
Weight (kg ± S.D.)	49.6 ± 10.3	46.6 ± 10.7	52.3 ± 9.1	<0.001	−8.37, −3.13
Length of Time in Nursing Home (months ± S.D.)	45.9 ± 12.3	47.5 ± 43.9	44.4 ± 46.2	0.632	−8.75, 17.84
	(range: 1–228)				
Estimated Length of Time on Tube Feeding					
<1 month	5 (2.1)	3 (1.3)	2 (0.8)		
1 to <3 months	6 (2.5)	2 (0.8)	4 (1.7)		
3 to <6 months	11 (4.6)	2 (0.8)	9 (3.8)		
6 to <12 months	15 (6.3)	6 (2.5)	9 (3.8)	0.072	
12 to <36 months	64 (26.7)	31 (12.9)	33 (13.8)		−
≥36 μοντησ	133 (55.4)	63 (26.3)	70 (29.2)		
Unsure	6 (2.5)	6 (2.5)	0 (0.0)		
Feeding Mode					
NG	234 (97.5)	111 (46.3)	123 (51.2)	0.687	−
PEG	6 (2.5)	2 (0.8)	4 (1.3)		
Types of Feeds					
Standard	126 (52.5)	54 (47.8)	72 (56.7)		
High Calorie & Protein	34 (14.2)	15 (13.3)	19 (15.0)		
Fibre-Containing	11 (4.6)	5 (4.4)	6 (4.7)		−
Diabeties-Specific	58 (24.2)	34 (30.1)	24 (18.9)	0.387	
Specialized	11 (4.6)	4 (4.4)	6 (4.7)		
Estimated Caloric Intake^a^					
kCal/d (± S.D.)	1298 ± 305	1199 ± 295	1384 ± 287	<0.001	−261, −108
Actual Caloric Intake					
kCal/d (± S.D.)	1389 ± 301	1294 ± 250	1470 ± 319	<0.001	−251, −101
kCal/kg/d (± S.D.)	29.1 ± 8.4	29.0 ± 8.0	29.2 ± 8.9	0.236	−2.0, 2.4
Estimated Protein Intake^b^					
g/d (± S.D.)	59.5 ± 12.3	56.0 ± 12.9	62.7 ± 10.9	<0.001	−10.1, −3.8
Protein Intake					
g/d (± S.D.)	57.2 ± 16.5	54.1 ± 13.9	60.0 ± 18.1	0.007	−10.1, −1.6
g/kg/d (± S.D.	1.20 ± 0.41	1.22 ± 0.41	1.18 ± 0.41	0.534	−0.07. 0.14

NG: Nasogastric, PEG: Percutaneous Endoscopic Gastrostomy, 95% CI: 95^th^ Percentile Confidence Interval. ^a^ based on a 25-30kcal/kg/d requirement, ^b^ based on a 1.2g/kg/d requirement.

**Table 4:** Monthly (28-day) Cost of Full Enteral Nutrition in LTCF (*n* = 232). Prices Reported in Singapore Dollars (Median with IQR).

Cost Component Per Resident	Standard Price	30% Discounted Price	45% Discounted Price
Cost of Feeds Per Month	325.81 (265.44, 386.69)	189.73 (138.10, 245.71)	185.50 (138.10, 222.60)
Cost of Nursing Per Month	503.37 (503.37, 503.37)	NA	NA
Cost of HEN Accessories (Feeding Tube, Syringes) Per Month	35.30 (35.30, 35.30)	NA	NA
Total Cost of HEN Per Month			
All Feed Types (*n* = 232)	856.19 (804.11, 955.79)	726.49 (655.49, 804.32)	715.85 (653.75, 776.72)
Ready-To-Use (*n* = 108)	882.68 (827.34, 1000.43)	765.24 (677.55, 848.36)	724.17 (658.45, 829.48)
Powdered (*n* = 124)	805.79 (794.32, 901.45)	692.11 (653.75, 776.73)	692.11 (653.75, 776.73)
Standard (*n* = 126)	855.57 (804.11, 957.16)	689.38 (653.75, 776.73)	688.27 (653.75, 776.73)
High Calorie & Protein (*n* = 38)	864.11 (793.53, 960.20)	749.37 (639.69, 812.56)	701.34 (628.44, 779.58)
Specialized (*n* = 68)	860.81 (733.49, 949.84)	773.15 (728.51, 866.44)	756.79 (713.60, 866.44)

NA: Not Applicable. Standard supplements include Polymeric 1.0 kcal/mL supplements. High Calorie & Protein supplements include 1.2–2.0 kcal/mL supplements. Specialized supplements include Diabetes-Specific, Renal and Peptide-Based supplements. Assumption of one change of feeding syringe per week was made, as per usual nursing home practices.

## Appendix A. Description of Types of LTCF in Singapore

**Table A1:** Description of LTCFs in Singapore (based on 2018 data from https://www.moh.gov.sg/resources-statistics/singapore-health-facts/health-facilities).

	Category of Nursing Homes	Description
**1**	Public or Voluntary Welfare Organizations’ (VWO) or Not-For-Profit Nursing Homes (*n*= 44)	Public LTCF refer to those owned/controlled by a government unit or another public corporation. Not-for-profit LTCF refer to those providing health services but not permitted to be a source of income, profit or financial gain. Singapore Citizens/Permanent Residents who meet the means-test criteria are referred through Agency for Integrated Care (AIC) to VWO Nursing Homes. May or may not receive Ministry of Health (MOH) subsidies to fund up to 75% of the fees payable by patients. For those that do not receive MOH subsidies – may be funded through the Home’s own fund-raising efforts and patients’ fees. The VWOs may provide additional support through Medifund (A Government Endowment Fund for the needy patients) and VWO welfare fund if the patients require further financial and social assistance.
**2**	Private Nursing Homes which are under MOH portable subsidy scheme (*n*= 21)	Private LTCF refer to those for the purpose of providing health services and are capable of generating a profit or financial gains. The MOH has extended subsidies to patients admitted to accredited private homes who meet the means-test criteria since 2003. A certain proportion of their beds are set aside for residents who are eligible for MOH subsidies and referred through AIC. This scheme allows for more private homes to participate in the provision of MOH-subsidised care.
**3**	Private Nursing Homes which are not under the MOH portable subsidy scheme (*n*= 7)	Private LTCF refer to those for the purpose of providing health services and are capable of generating a profit or financial gains. Self-admission or referral. For full fee paying patients

AIC: Agency of Integrated Care - the main coordinating body for the support of aged care services in Singapore, LTCF: Long-Term Care Facilities, MOH: Ministry of Health, VWO: Voluntary Welfare Organisation.

## Appendix B. Survey Form

**Part 1.** Name of Nursing Home: ________________________________ (This will not be recorded during data analysis).

Participant completing this survey:

Name: _________________ Role in Nursing Home: __________________ (This will not be recorded during data analysis).

**Description of Nursing Home**	
Total Number of Beds in the Home (Answer all questions)	(a) Total number of beds _______ (b) Month of ____ 2018 (e.g. Jun, Jul or Aug)
Total Number of Residents in the Home (Answer all questions)	(a) Number of residents _______ (b) Month of _____ 2018 (e.g. Jun, Jul or Aug)
Number of Dietitians(Answer where applicable only)	(a) Full-time staff of Nursing Home (*e.g. 1, 2 or 3*): ________ OR (b) Part-time staff of Nursing Home (*e.g. 1, 2 or 3*): ________ OR (c) Dietetic service bought from other hospitals/ private practices (*e.g. 1, 2 or 3*): ________
If Part-time dietitian or Outsourced ^#^ Dietetic Services (Answer where applicable only)	(a) Number of visits per month: _______ OR (b) Number of visits per quarter (3 months): _______ OR (c) Number of visits per half yearly (6 months): ________
Frequency of dietetic review for each tube-fed patient (Tick and answer where applicable only)	How soon is resident reviewed on admission to the Home *(More than 1 answer is possible)*: □ Automatic referral on admission with fixed review once every _____ month(s) □ Automatic referral on admission but follow-ups dependent on Dietitian’s order □ If Hospital Memo or Transfer Letter requested □ If Doctor-in-charge in the Home requested □ If Family Member(s) requested □ If Nursing Staff requested as resident is eating poorly
Nutritional Screening in Nursing Home	
Is nutritional screening performed for all patients?	□ Yes □ No
Is nutritional screening for all patients repeated?	□ Yes □ No if Yes, how frequent is it repeated: _________________ *(e.g. once a month, once every 3 months)*
Number of Speech Therapists	(a) Full-time staff of Nursing Home (*e.g. 1, 2 or 3*): ________ OR (b) Part-time staff of Nursing Home (*e.g. 1, 2 or 3*): ________ OR (c) Therapist service bought from other hospitals/ private practices (*e.g. 1, 2 or 3*): _______
If Part-time Speech Therapist or Outsourced^#^ Therapy Services	(a) Number of visits per month: _______ OR (b) Number of visits per quarter (3 months): _______ OR (c) Number of visits per half yearly (6 months): ________
Prevalence and Incidence of Dysphagia (Swallowing Impairment on Modified Textured Diet)	
Number of residents with dysphagia or swallowing impairment	(a) Number of residents diagnosed with dysphagia, placed on modified texture (e.g. pureed, blended, minced) diet or thickened fluids this month: ____________ (b) TOTAL number of residents diagnosed with dysphagia or who are placed on modified texture (e.g. pureed, blended, minced) diet or thickened fluids: ___________________
Prevalence and Incidence of Enteral Nutrition (ALL Types of Tubes Feeding)	
Number of residents on Enteral Nutrition (Tube Feeding)	(a) Number of residents with newly inserted feeding tube *this month*: _______ (b) TOTAL number of residents with feeding tubes: _______
Type of Enteral Nutrition Delivery Mode	
Types of Feeding Tubes (please indicate NA if there are no patients on any of types of feeding tubes)	Number of residents on (a) Nasogastric Tube (NGT): _____ (b) Nasojejunal Tube (NJT): _____ (c) Percutaneous Endoscopic Gastrostomy (PEG): _____ (d) Percutaneous Endoscopic Jejunostomy (PEJ): _____ (e) Feeding Jejunostomy (FJ): _____
How are feeds delivered (please indicate NA if there are no patients on any particular delivery method)	Number of residents on (a) Bolus feeding: _____ (b) Continuous feeding with pump: ______ (c) Continuous feeding via gravity drip: ______
^#^ Outsourced from private sector or government hospitals.	

**Resident Demographics**								
Patient Number	Age (Years)	Gender (F/M)	Weight (kg)	Current Feeding Regimen *(e.g. Ensure 6 scoops x 6 feedings per day, water flushes not required)*	Full (F) or Supplemental (S) Feeding	Estimated Length of Time on Tube Feeding	Resident in Home Since Month/Year *(e.g.Aug/ 2018)*	Feeding Mode (NG/ NJ/ PEG/ FJ)
						A = < 1 month		
						B = 1 to < 3 months		
						C = 3 to < 6 months		
						D = 6 to < 12 months		
						E = 12 to < 36months (3 years)		
						F = > 36 months (3 years)		
						G = Unsure		
E.g.	80	F/M	55	Ensure 7 scoops x 2 feedings	S	D	Jun 2016	NG/ NJ/ PEG/ FJ
1		F/M						NG/ NJ/ PEG/ FJ
2		F/M						NG/ NJ/ PEG/ FJ
3		F/M						NG/ NJ/ PEG/ FJ
4		F/M						NG/ NJ/ PEG/ FJ
5		F/M						NG/ NJ/ PEG/ FJ
6		F/M						NG/ NJ/ PEG/ FJ
7		F/M						NG/ NJ/ PEG/ FJ
8		F/M						NG/ NJ/ PEG/ FJ
9		F/M						NG/ NJ/ PEG/ FJ
10		F/M						NG/ NJ/ PEG/ FJ
11		F/M						NG/ NJ/ PEG/ FJ
12		F/M						NG/ NJ/ PEG/ FJ
13		F/M						NG/ NJ/ PEG/ FJ
14		F/M						NG/ NJ/ PEG/ FJ
15		F/M						NG/ NJ/ PEG/ FJ

**Part 2.**

**Type of Enteral Feeds Available in the Home**	
Feeds available in the nursing home	Please indicate ALL the feeds CURRENTLY available in your Home (*e.g. Ensure Powder, Resource Plus*): __________________________________________ __________________________________________ __________________________________________ __________________________________________
Other Components. (Please indicate NA if not applicable)	(a) How often are nasogastric feeding tubes changed *(e.g. once a month)*: ___________________ (b) How much time (minutes) does it take to change a nasogastric feeding tube (*estimate*): ___________ (c) For residents on continuous/gravity feeding, how often are feeding bags changed: ________ (d) Can special feeds (e.g. renal feeds or high calories feeds) not usually available in your Home be ordered in for the resident if requested/required? □ Yes □ No □ Not Sure
Manpower / Nursing Time	
Number of Staff *(Please indicate NA if not applicable)*	(a) **Total Number** of Staff Nurses in the Home: ________________ (b) Number of Staff Nurses **per shift**: ________________________ (c) **Total Number** of Enrolled Nurses: _______________________ (d) Number of Enrolled Nurses **per shift**: _____________________ (e) **Total Number** of Nursing Aides: _________________ (f) Number of Nursing Aides **per shift**: _______________ (g) Nursing to Patient Ratio (*e.g. 1 Staff Nurse: 10 patients*): __________
Feeding Administration	Who performs the enteral feeding? (*please tick all that is applicable*) □ Staff Nurse □ Enrolled Nurse □ Nursing Aide How long does it take to perform ONE enteral tube feeding? (*Please give an estimate e.g. 10min, 15min, 20min*) __________________________________________ Do you think that continuous feeding (with pump) should be made available in nursing homes? □ Yes □ No □ Not Sure
Training	How many hours of training on Nutrition Support do Nursing Staff receive per year? □ < 1 h □ 1 to < 3 h □ 3 to < 5 h □ ≥ 5 h As a Healthcare Professional in a Nursing Home, do you think you are provided with adequate education/training in Nutritional Support for your residents? □ Yes □ No □ Not Sure
